# A Complicated Case of Obstetrics

**Published:** 1898-03

**Authors:** I. J. Clemons

**Affiliations:** Monaville, Texas


For the Texas Medical Journal

### A Complicated Case of Obstetrics.

BY I. J. CLEMONS, M. D., MONAVILLE, TEXAS.

On December 29th, 1897, I was called to see Mary F., colored, aet. 21, primipara, who had been in labor about twenty hours. According to her statement the pregnancy had advanced to the thirty-eighth week. She further stated that she had received a severe fall three days prior, and that she had felt no fœtal movements since shortly after the fall.

I could not detect any fœtal heart sound with the stethoscope, nor could I make out a diagnosis by abdominal palpation. Upon vaginal examination the breech was found to present in the second (S. D. A.) position.

Uterine contractions were fairly good, but were not sufficient to advance the progress of labor in the least. After administering sulphate of quinine, grs. xx, the pains greatly increased; but there was still no advance toward the termination of labor. I then brought down a foot, upon which followed closely the delivery of the body. In spite of my guarding against it by keeping the dorsum anterior, the occiput rotated posterior. The cord was pulseless; and after rotating occiput to the front by means of two fingers of right hand in vagina, and with the left hand externally manipulating through the abdominal wall, I soon succeeded in completing the delivery of a still-born child.

The continued large size of the uterus led to the suspicion of a multiple pregnancy, and upon examination this state of affairs was evidenced. The second fœtus was found to be in the transverse position. The uterus still being relaxed, I managed to turn it into a vertex position by Braxton Hicks' (bimanual) method. As the head would not advance after engaging in O. L. A. position, I found per vaginal examination to my great disappointment that the left arm was turned back around the neck. The fœtus being forcibly impacted in the pelvis, and the matter being in a somewhat collapsed condition, I decided upon mutilation of the fœtus after finding that there was no fœtal heart sound to be detected. The administration of chloroform and turning was decided to be a too hazardous operation with the mother in such collapsed condition, and that craniotomy would be much safer for the mother. After having removed the bones of the vault and its contents, I succeeded in getting hold of the arm, which I amputated at the elbow. Protecting the exposed base of skull by the scalp flap, to avoid injury to the maternal parts, delivery of the second fœtus was soon accomplished by gentle traction. The patient being now in an almost pulseless condition I administered strychnia sulph. gr. $\frac{1}{30}$; tr. digitalis, gtts. xxx, hypodermically; rubbed limbs with whisky and applied hot water bottles to body. In thirty minutes I was agreeably surprised to find the pulse fallen to 90 per minute. The uterus being entirely exhausted, the placenta had to be removed manually amidst profuse hemorrhages, which latter was held under control by bi-manual compression of the uterus. The uterus was thoroughly irrigated with a 1:500 solution of carbolic acid, and the patient given two drachms of ergot and some brandy. The patient has never felt any outward symptoms since; temperature never went above 99½° at this date; about twenty days later the patient is up attending to her ordinary housework.

